# Apolipoprotein H induces sex-specific steatohepatitis and gut dysbiosis during chronic hepatitis B infection

**DOI:** 10.1016/j.isci.2023.106100

**Published:** 2023-02-01

**Authors:** Yaming Liu, Yangtao Wu, Xiaoming Jiang, Bo Chen, Jing Lu, Zexin Cai, Baorong Fu, Wei Zheng, Ruihong Wu, Gang Chen, Shulan Tian, Jianlin Ren

**Affiliations:** 1 Department of Gastroenterology and Hepatology, Xiamen University Zhongshan Hospital, Xiamen, Fujian Province 361001, China; 2 Department of Digestive Diseases, School of Medicine, Xiamen University, Xiamen, Fujian Province 361001, China; 3 Division of Computational Biology, Department of Quantitative Health Sciences, Mayo Clinic, Rochester, MN 55905, USA; 4 National Institute of Diagnostic and Vaccine Development in Infectious Disease, Xiamen University, Xiamen, Fujian Province 361001, China; 5 Department of Hepatology, The First Hospital of Jilin University, Changchun, Jilin Province 132001, China; 6 Department of Hepatobiliary Surgery, The first affiliated Hospital of Wenzhou Medical University, Wenzhou, Zhejiang Province 325000, China; 7 Department of Pathology, Xiamen University Zhongshan Hospital, Xiamen, Fujian Province 361001, China

**Keywords:** Virology, Human metabolism, Microbiome

## Abstract

Apolipoprotein H (APOH) is involved in lipid metabolism and functions as an acute-phase protein during hepatitis B virus (HBV) infection. Herein, we explored whether APOH acts on the development of fatty liver upon chronic HBV infection. Serum APOH level was significantly lower in cirrhosis patients than in healthy controls or patients with chronic infection. It showed sex bias, with elevated levels in female patients with chronic infection. Also, serum APOH levels were negatively correlated with HBV surface antigen (HBsAg) but positively correlated with albumin and triglyceride levels. In *In vitro* HBV infection model, HBV upregulated *APOH* expression in a non-temporal manner, and *HBsAg* levels were elevated by silencing *APOH*. RNA sequencing (RNA-seq) demonstrated bidirectional expression of *APOH,* which impacted the immunoregulation upon infection or the metabolic regulation in HepG2.2.15 cells. Then, *ApoH*^−/−^ mice with persistent HBV replication displayed steatohepatitis and gut microbiota dysbiosis with synergistic sex differences. Our study deciphers the roles of APOH in chronic liver diseases.

## Highlights

• Serum APOH levels are decreased and show sex bias during chronic HBV infection
• APOH performs a later regulatory role in metabolism during chronic HBV infection
• *ApoH*^−/−^ mice display spontaneous steatohepatitis with synergistic sex differences
• The gut microbiota is disordered in *ApoH*^−/−^ HBV mice in a sex-specific manner

## Graphical abstract

**Figure:**
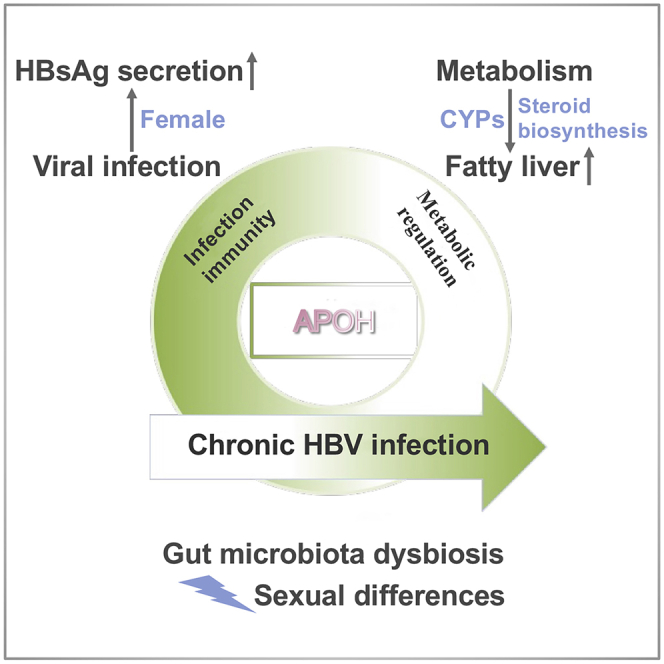

## Introduction

Chronic hepatitis B (CHB) virus infection remains an unresolved global public health problem, with more than 400 million people infected worldwide.^1^^,^^2^^,^^3^^,^^4^^,^^5^ Recently, multiple studies have revealed that approximately a quarter of CHB patients have concurrent steatosis. The potential consequences of developing non-alcoholic fatty liver disease (NAFLD) in patients with CHB infection include active hepatitis, progression of chronic liver disease, and the development of hepatocellular carcinoma.^6^^,^^7^^,^^8^^,^^9^ It has been reported that the gut microbiota acts as a major determinant of this relationship in the onset and clinical course of liver diseases and that sex is significantly associated with viral infection, lipid metabolism, and gut microbiota composition.^10^^,^^11^^,^^12^ It remains to be seen what mechanisms underlie the relationship between steatosis and chronic hepatitis B virus (HBV) infection.

It is well known that apolipoprotein H (APOH, also known as beta2-glycoprotein I) is an abundant plasma apolipoprotein primarily produced in the liver^13^^,^^14^ and is closely associated with lipid metabolism.^15^^,^^16^^,^^17^^,^^18^^,^^19^^,^^20^ For instance, APOH binds to lipoproteins and activates the lipoprotein lipase during triglyceride metabolism. Moreover, the distinct APOH protein isoforms alter the expression of apolipoprotein B (APOB), apolipoprotein A (APOA), high-density lipoprotein cholesterol (HDL-C), triglyceride (TG), and total cholesterol (TC). In addition, APOH is reportedly an acute-phase protein during HBV infection, and the HBV surface antigen (HBsAg) binds to APOH with high affinity.^21^^,^^22^^,^^23^ We previously found that HBV and their large surface antigens directly upregulate APOH expression, which inhibits HBsAg secretion and further induces hepatocellular ER stress.^24^^,^^25^ Therefore, we hypothesize that APOH might be a point of convergence between hepatocyte steatosis and HBV-related chronic liver diseases.

This study analyzed the dynamics of serum APOH levels, by which we aim to understand the association between APOH and liver function in different disease conditions in patients with chronic HBV infection. Furthermore, we analyzed RNA sequencing (RNA-seq) data from HepG2.2.15 cells silencing or overexpressing *APOH*. We further constructed an *ApoH* gene-knockout mouse model and a persistent HBV replication mouse model to investigate the associations between APOH levels and hepatocyte steatosis, gut microbiota dysbiosis, and HBV infection. The study provides novel insights into the potential regulatory role of APOH in chronic HBV infection and its association with hepatocyte steatosis and gut microbiota dysbiosis.

## Results

### Analysis of serum APOH levels in patients with HBV-related chronic liver diseases

**Figure 1:**
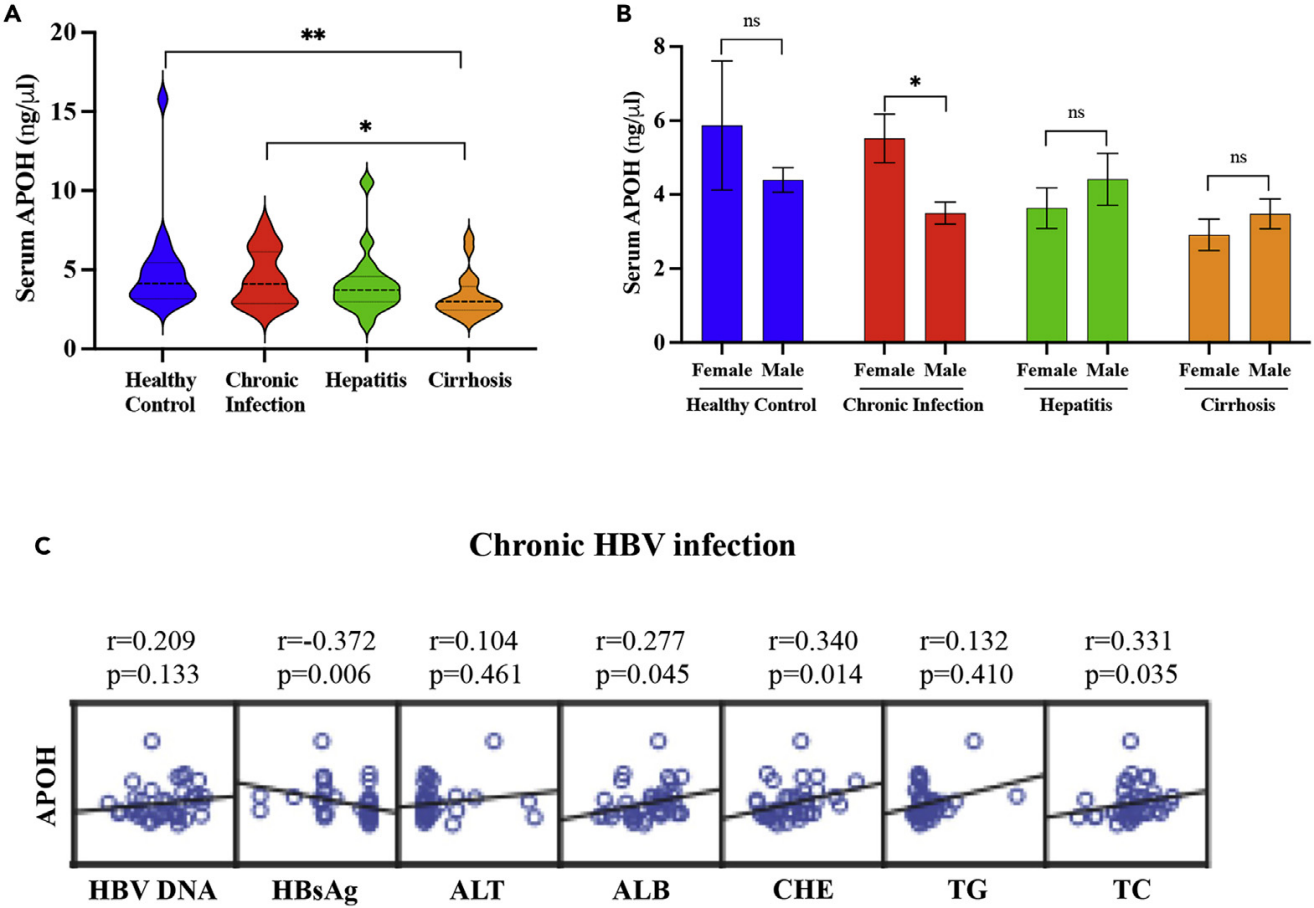
Analysis of serum APOH levels in patients with HBV-related chronic liver diseases (A) Serum APOH levels were detected using ELISA in patients with different statuses of chronic HBV infection. (B) Sex-dependent differences in serum APOH levels during chronic infection. Data are represented as mean ± SEM, ∗p < 0.05. (C) Correlation of APOH levels with viral load, liver function, and lipid levels in HBV-related chronic liver diseases.

The clinical and demographic information of this cohort is presented in Table 1. The serum APOH levels were significantly lower in patients with cirrhosis than those in healthy controls or patients with chronic infection (p < 0.05) (Figure 1A). In the chronic infection group, female patients had higher serum APOH levels than male patients (p < 0.05) (Figure 1B). Furthermore, while serum APOH levels were negatively correlated with HBsAg levels in patients, they were positively associated with albumin, cholinesterase, and TG levels (Figure 1C) (p < 0.05).

**Table 1:** Demographic data and serum markers of patients

	Healthy Control	Chronic Infection	Hepatitis	Cirrhosis	p value
Number	18	17	17	19	–
Gender (F/M)	7/11	8/9	6/11	5/14	0.647
Age in years	46.1 ± 13.5	41.5 ± 11.4	35.5 ± 10.8	51.1 ± 10.9	0.001
log10 HBVDNA	–	6.678 ± 1.919	6.465 ± 1.485	5.160 ± 2.026	0.033
log10 HBsAg	–	1.273 ± 0.375	1.389 ± 0.791	2.391 ± 0.040	0.000
log10 pre-S1	–	1.061 ± 0.439	0.270 ± 0.572	–	0.027
ALT (U/L)	20.944 ± 4.684	32.471 ± 13.459	296.118 ± 460.364	58.316 ± 32.263	0.000
AST (U/L)	17.667 ± 7.420	29.941 ± 13.184	399.706 ± 421.866	51.263 ± 30.621	0.001
ALB (g/L)	43.828 ± 3.356	40.106 ± 4.516	37.771 ± 5.209	30.653 ± 7.169	0.000
ALP (U/L)	57.222 ± 11.471	76.706 ± 33.522	96.294 ± 45.336	106.632 ± 39.664	0.001
GGT (U/L)	21.444 ± 7.524	39.706 ± 41.311	112.412 ± 101.245	123.421 ± 162.172	0.005
TBIL (μmol/L)	10.500 ± 0.000	14.400 ± 5.690	80.700 ± 126.182	44.574 ± 58.994	0.121
DBIL (μmol/L)	3.400 ± 0.000	4.271 ± 1.789	42.024 ± 68.281	20.853 ± 32.694	0.097
IBI (μmol/L)	7.100 ± 0.000	10.129 ± 4.088	39.188 ± 61.973	23.721 ± 27.349	0.188
CHE (U/L)	–	7836.353 ± 3151.527	6641.188 ± 2314.363	4291.842 ± 1852.798	0.000
TBA (μmol/L)	2.200 ± 0.000	11.106 ± 17.465	79.513 ± 119.825	67.432 ± 97.351	0.128
TG (mmol/L)	1.046 ± 0.351	1.101 ± 0.524	1.785 ± 1.864	0.944 ± 0.450	0.088
TC (mmol/L)	4.352 ± 0.503	4.109 ± 0.645	3.707 ± 0.718	3.525 ± 0.705	0.002
HDL(mmol/L)	1.344 ± 0.233	1.518 ± 0.416	1.088 ± 0.650	1.234 ± 0.491	0.172
LDL(mmol/L)	2.586 ± 0.417	2.361 ± 0.623	2.264 ± 0.700	2.095 ± 0.522	0.070
Platelet (10ˆ9/L)	225.778 ± 46.225	178.500 ± 55.798	171.889 ± 61.655	85.105 ± 47.838	0.000
AFP (ng/mL)	5.978 ± 0.668	2.275 ± 1.011	18.845 ± 23.980	58.286 ± 131.693	0.665

### HBV stimulation upregulates *APOH* in NTCP-reconstituted HepG2 cells

**Figure 2:**
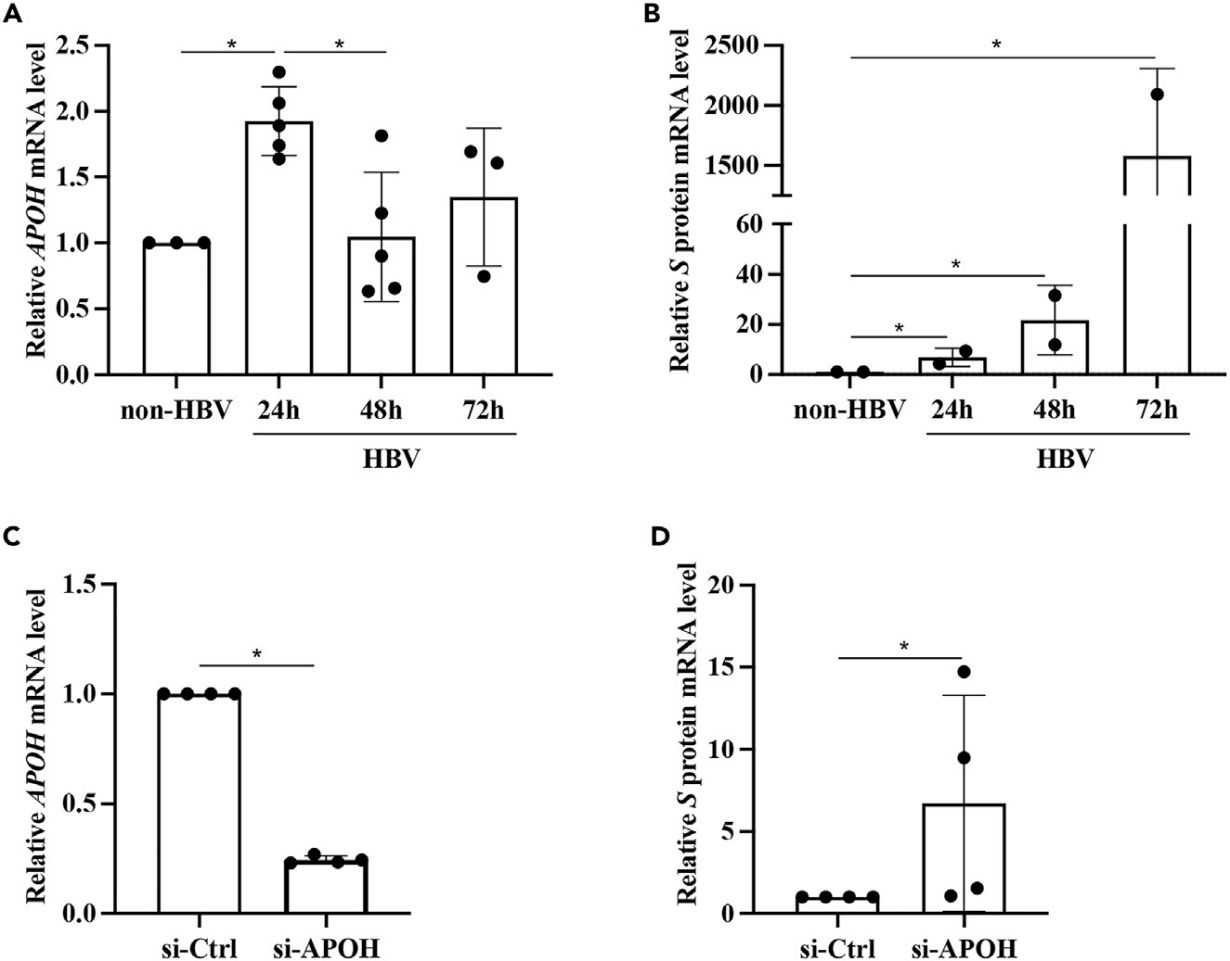
HBV stimulation upregulates *APOH* in NTCP-reconstituted HepG2 cells (A) HepG2-NTCP-tet cells were seeded in 24-well plates and treated with HBV (100 ng/mL) for 24, 48, and 72 h. *APOH* mRNA levels were assayed using qPCR. (B) HBV *S* domain expression assayed using qPCR. (C and D) *APOH* was silenced in cells through transfection with a small interfering RNA (siRNA). A control group (siControl) was included. After 48 h, the cells were harvested, and the total mRNA was extracted for RNA-seq. Data are represented as mean ± SEM, ∗p < 0.05.

To confirm whether the stage of HBV infection impacts *APOH* expression levels, we used an *in vitro* HBV infection system, i.e., sodium taurocholate co-transporting polypeptide (NTCP)-reconstituted HepG2 cells. We confirmed that HBV temporally upregulated *APOH* mRNA expression in the early stage of HBV infection (Figure 2A). Figure 2B showed the levels of HBV *S* mRNA in NTCP-reconstituted HepG2 cells after HBV infection. Further experimentation indicated that *APOH* gene knockdown (Figure 2C) upregulated HBV *S* mRNA expression (Figure 2D).

### Variable *APOH* expression reveals changes in enriched signaling pathways and alters the expression of metabolic regulatory genes

**Figure 3:**
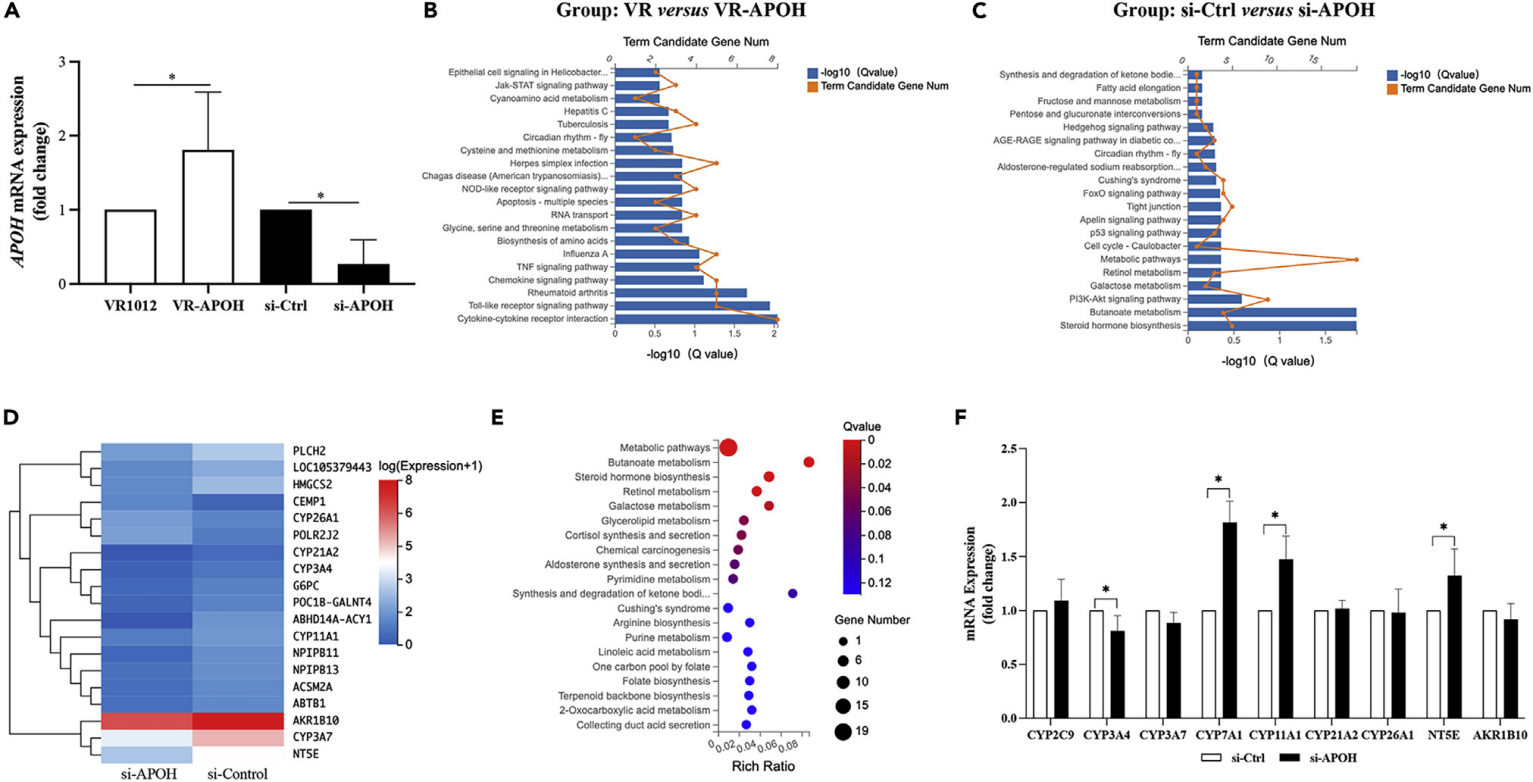
Variable *APOH* expression reveals changes in enriched signaling pathways and alters the expression of metabolic regulatory genes HepG2.2.15 cells were cultured overnight in a 6-well plate and transfected with siRNA against *APOH* or VR-APOH-myc or a control vector. After 48 h, the cells were harvested, and the total mRNA was extracted for RNA-seq. (A) *APOH* mRNA levels were assayed using qPCR; ∗p < 0.05. (B) The KEGG pathway enrichment of the total differentially expressed genes (DEGs) for *APOH* overexpression group versus the control group. (C) The KEGG pathway enrichment of total DEGs for the *APOH*-silenced versus control group. (D) Cpmparison of DEGs between the *APOH*-silenced group and the control group. (E) A bubble diagram providing the details regarding the metabolic pathways from the KEGG pathway analysis. (F) Relative expression of genes involved in regulating CYP450 enzymes in the *APOH*-silenced and control groups. Data are presented as the mean ± SEM, ∗p < 0.05.

*APOH* was expressed at the mRNA level in different groups (Figure 3A). We consulted the Kyoto Encyclopedia of Genes and Genomes (KEGG) pathways to analyze which signaling pathways were affected by *APOH* expression. In cells overexpression *APOH*, the enriched signaling pathways were mainly cytokine-cytokine receptor interactions and Toll-like receptor signaling pathway (Figure 3B). However, in *APOH*-silenced cells, metabolic pathways were enriched based on KEGG pathway analysis (Figure 3C). A list of 19 differentially expressed genes (DEGs) between the two groups is shown in Figure 3D. Moreover, the analyzed metabolic regulatory pathways are illustrated in the bubble diagram (Figure 3E).

Notably, cytochrome P450 (CYP) enzyme-related genes were over-represented in the 19 DEGs, including *CYP3A4*, *CYP3A7*, *CYP7A1*, *CYP11A1*, *CYP21A2*, and *CYP26A1*. CYP450 enzymes have been well documented to act as a terminal oxidase in the multi-function oxidase system to metabolize different endogenous substrates and xenobiotics (Table S2).^26^^,^^27^ The isoenzymes CYP7A1, CYP11A1, and CYP21A2 primarily participate in steroid biosynthesis and metabolism. Of the nine DEGs examined (Figure 3F), *CYP7A1*, *CYP11A1*, and *NT5E* showed significantly increased expression in the *APOH*-silencing group (p < 0.05), while *CYP3A4* was sharply decreased in the same group (p < 0.05). Therefore, we conclude that APOH production is decreased in the background of chronic HBV infection, which activates metabolism pathways and mainly affects steroid hormone biosynthesis.

### *APOH*-knockout mice exhibit signs of liver damage and steatohepatitis

**Figure 4:**
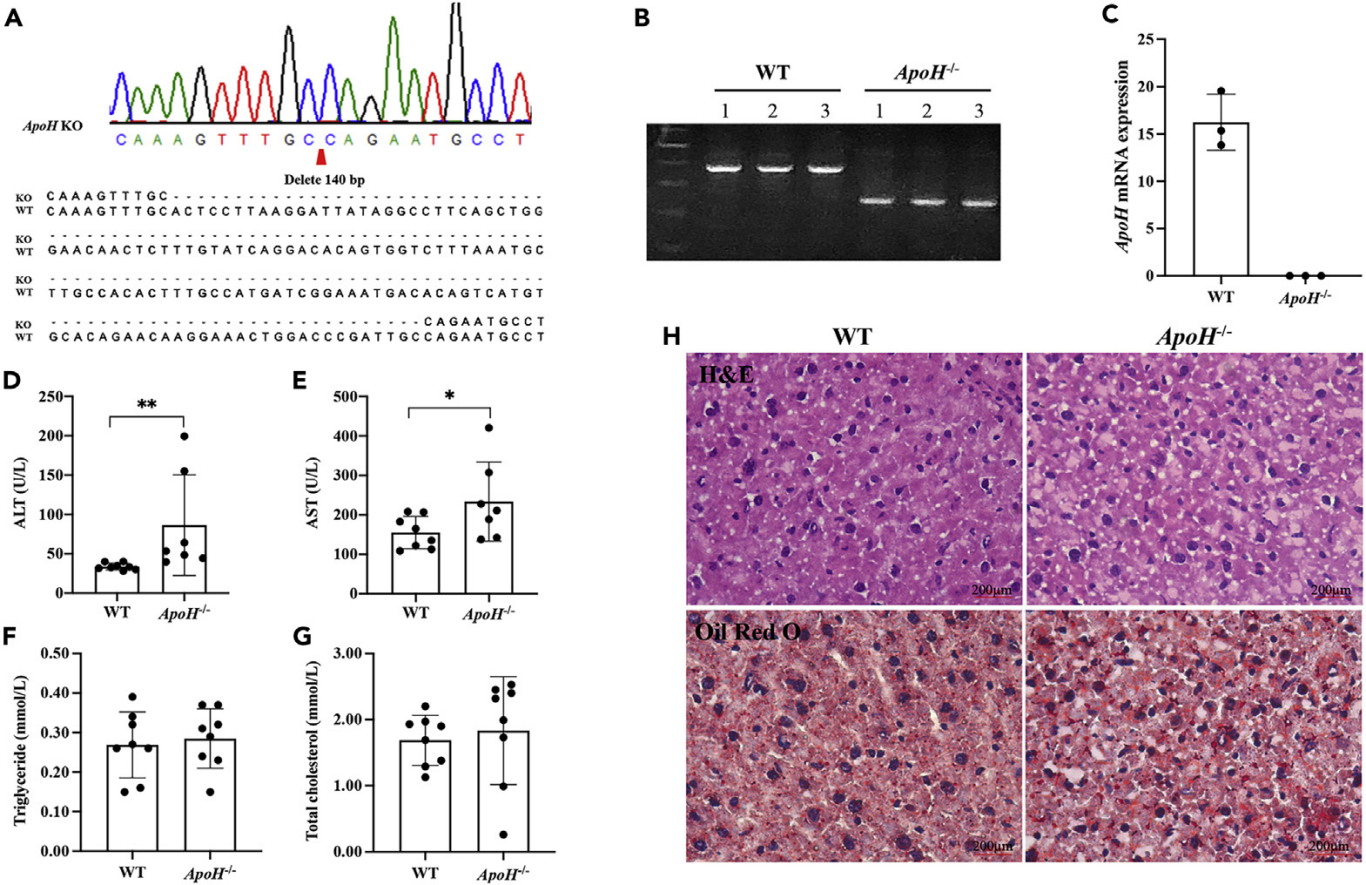
*ApoH*-knockout mice exhibit signs of liver damage and steatohepatitis (A) C57BL/6J mice were used to generate an *ApoH*-knockout mouse model using CRISPR, in which 140 bp were deleted from transcriptional X1 exon 3. (B) The PCR products were resolved on a 1.7% agarose gel for genotyping. (C) RNA was isolated from the liver tissues of wild-type (WT) and *ApoH*^−/−^ mice. *ApoH* expression at the mRNA level was detected using qPCR. (D–G) Eight- to ten-week-old mice were sacrificed to detect serum levels of alanine aminotransferase (ALT), aspartate aminotransferase (AST) (D and E), triglyceride, and total cholesterol (F and G) levels. (H) Hematoxylin and eosin (H&E) and Oil Red O-stained liver tissues (20X). Data are presented as the mean ± SEM, ∗p < 0.05.

Next, we constructed an *ApoH*-knockout mouse model to investigate the potential function of APOH in the liver. The 6-week *ApoH*^−/−^ mice were used for genotype identification. A part of the sequencing data is shown in Figure 4A, and the PCR products were resolved on an agarose gel (Figure 4B). *ApoH* mRNA expression was highly reduced, confirmming the knockdown efficiency (Figure 4C). The serum alanine aminotransferase (ALT) and aspartate aminotransferase (AST) levels were significantly higher in *ApoH*^−/−^ mice than in wild-type (WT) mice (p < 0.05) (Figures 4D and 4E), but no significant differences were observed for TG and TC levels (p > 0.05) (Figures 4F and 4G). We also found apparent steatosis in the liver sections of *ApoH*^−/−^ mice stained with H&E or Oil Red O (Figure 4H). The results suggested that the downregulation of *ApoH* expression induced mouse spontaneous steatohepatitis.

### *ApoH*^−/−^ mice display altered microbiota diversity and differentially abundant bacterial species

**Figure 5:**
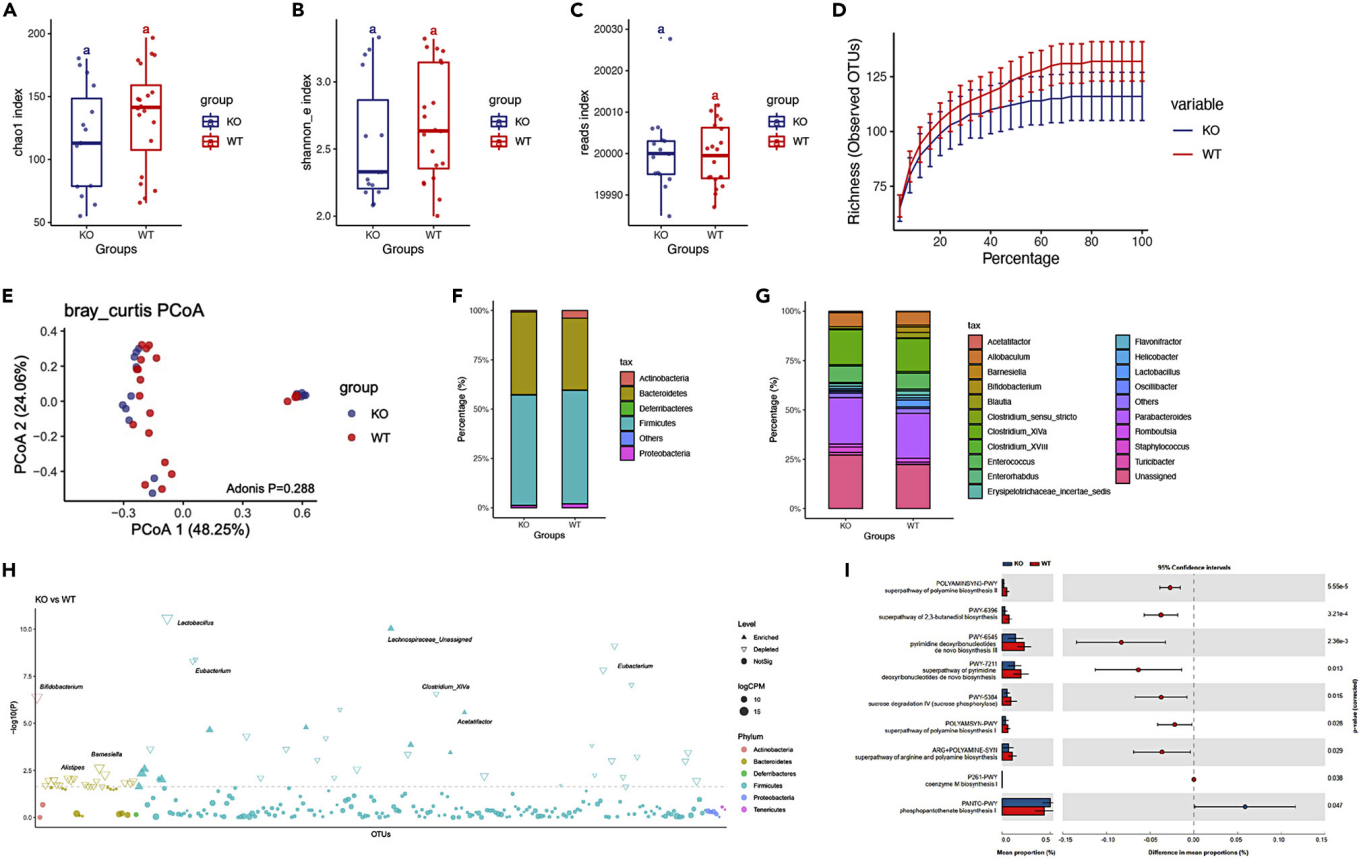
*ApoH*^−/−^ mice display altered microbiota diversity and differentially abundant bacterial species Alpha diversity of WT and *ApoH*^−/−^ mice, comprising the (A) Chao1 index, (B) Shannon-Weiner biodiversity index (Shannon index), and (C) Reads index; p > 0.05. (D) Beta diversity of WT and *ApoH*^−/−^ mice (p > 0.05). (E) Principal coordinate analysis (PCoA) of the beta diversity comparison, using Bray Curtis distances, revealing the separation of microbial communities based on *ApoH*-knockout genotype (p > 0.05). Bacterial composition at the phylum (F) and genus (G) levels in WT and *ApoH*^−/−^ mice. (H) Manhattan diagram showing differentially abundant bacterial species in *ApoH*^−/−^ mice compared with that in WT mice. (I) Predictive functional profiling of microbial communities using PICRUSt MetaCys pathways.

We explored the gut community diversity and the difference in bacterial species abundance between *ApoH*^−/−^ and WT mice. The 16S rRNA sequencing data indicated no significant difference in the alpha and beta diversities between the two mice groups (p > 0.05) (alpha diversity: Figures 5A–5C, beta diversity: Figures 5D and 5E). We further analyzed the bacterial composition at the phylum and genus levels (Figures 5F–5H). The total bacterial composition and abundance were reduced in *ApoH*^−/−^ mice compared to those in the WT mice. In *ApoH*^−/−^ mice, at the phylum level, the abundance of Bacteroidetes was increased, while that of *Firmicutes* and *Actinobacteria* was sharply decreased. At the genus level, the abundance of *Lachnospiraceae, Parabacteroides, Staphylococcus, and Acetatifactor* increased, in contrast to the decrease in abundance for *Lactobacillus, Bifidobacterium*, *ClostridiumXIVa*, *Eubacterium*, and *Barnesiella*.

Next, to investigate potential metabolic differences between *ApoH*^−/−^ and WT mice, we performed the predictive functional profiling of microbial communities using the PICRUSt MetaCyc pathway (Figure 5I). The activity of phosphopantothenate biosynthesis I (PANTO-PWY) pathway was higher in *ApoH*^−/−^ mice (p < 0.05). The downregulated pathways in *ApoH*^−/−^ mice included the following: superpathways of polyamine biosynthesis II, 2,3-butanediol biosynthesis, *de novo* pyrimidine deoxyribonucleotides biosynthesis, and polyamine biosynthesis I, along with *de novo* pyrimidine deoxyribonucleotides biosynthesis III, sucrose degradation IV (sucrose phosphorylase), and coenzyme M biosynthesis I.

### *ApoH*^−/−^ mice with persistent HBV infections exhibit increased levels of HBsAg secretion and liver steatosis

**Figure 6:**
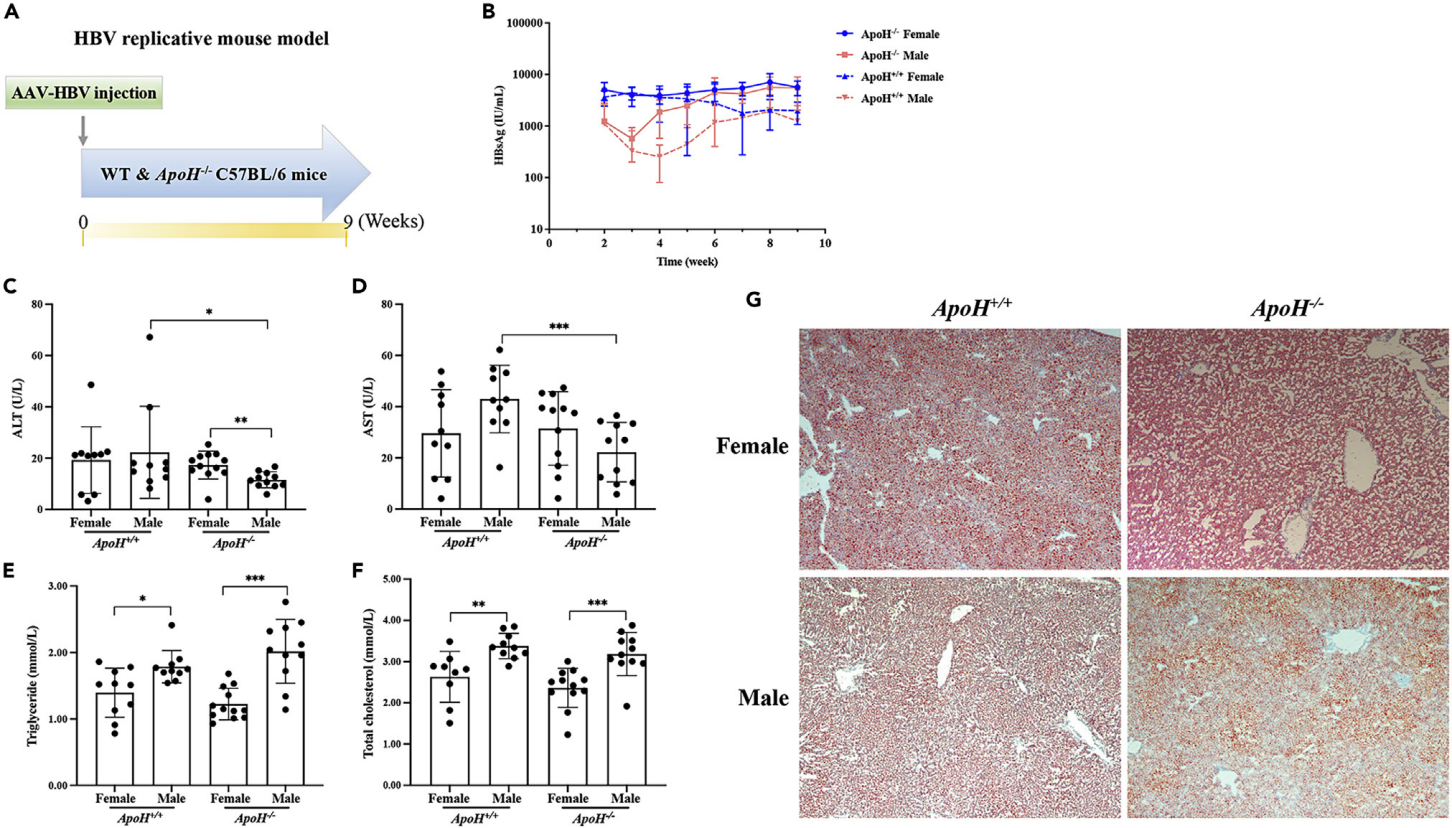
*ApoH*^−/−^ mice with persistent HBV infections exhibit increased levels of HBsAg secretion and liver steatosis (A) Generation of a persistent HBV replication mouse model using WT and *ApoH*^*−/−*^ C57BL/6J mice. The plasmid rAAV8-1.3HBV (5 × 10^10^ μg/200 μL per mouse) was injected into the tail vein, and orbital venous blood was collected weekly until 9 weeks after injection. (B) Serum HBsAg titers were measured using ELISA. Model mice were sacrificed to detect serum ALT, AST (C and D), triglyceride, and total cholesterol (E and F) levels. (G) Image of H&E- and Oil Red O-stained liver tissues (20X). Data are presented as the mean ± SEM, ∗p < 0.05.

The HBV replication mouse model was generated using six- to seven-week-old *ApoH*^*−/−*^ mice and C57BL/6 WT mice as controls (Figure 6A). The mouse model was used to study the effects of APOH in the context of persistent HBV infection and immune tolerance. Serum HBsAg levels were higher in *ApoH*^−/−^ mice than in WT mice (Figure 6B). We also found that the HBsAg levels in female mice were higher than those in male mice after seven weeks of HBV infection (Figure S3). Serum ALT and AST levels in male *ApoH*^−/−^ mice were significantly lower than those in male WT mice (p < 0.05) (Figures 6C and 6D). The serum TG and TC levels were sharply increased in male mice compared with those in females, independent of APOH expression (p < 0.05) (Figures 6E and 6F). Furthermore, significant levels of hepatocyte steatosis were observed in *ApoH*^−/−^ mice with a persistent HBV infection (Figure 6G). Therefore, *ApoH* downregulation predominately promoted HBsAg secretion and induced steatohepatitis in female *ApoH*^−/−^ mice.

### Mice with persistent HBV replication have altered gut microbiota community diversity and differentially abundant bacterial species

**Figure 7:**
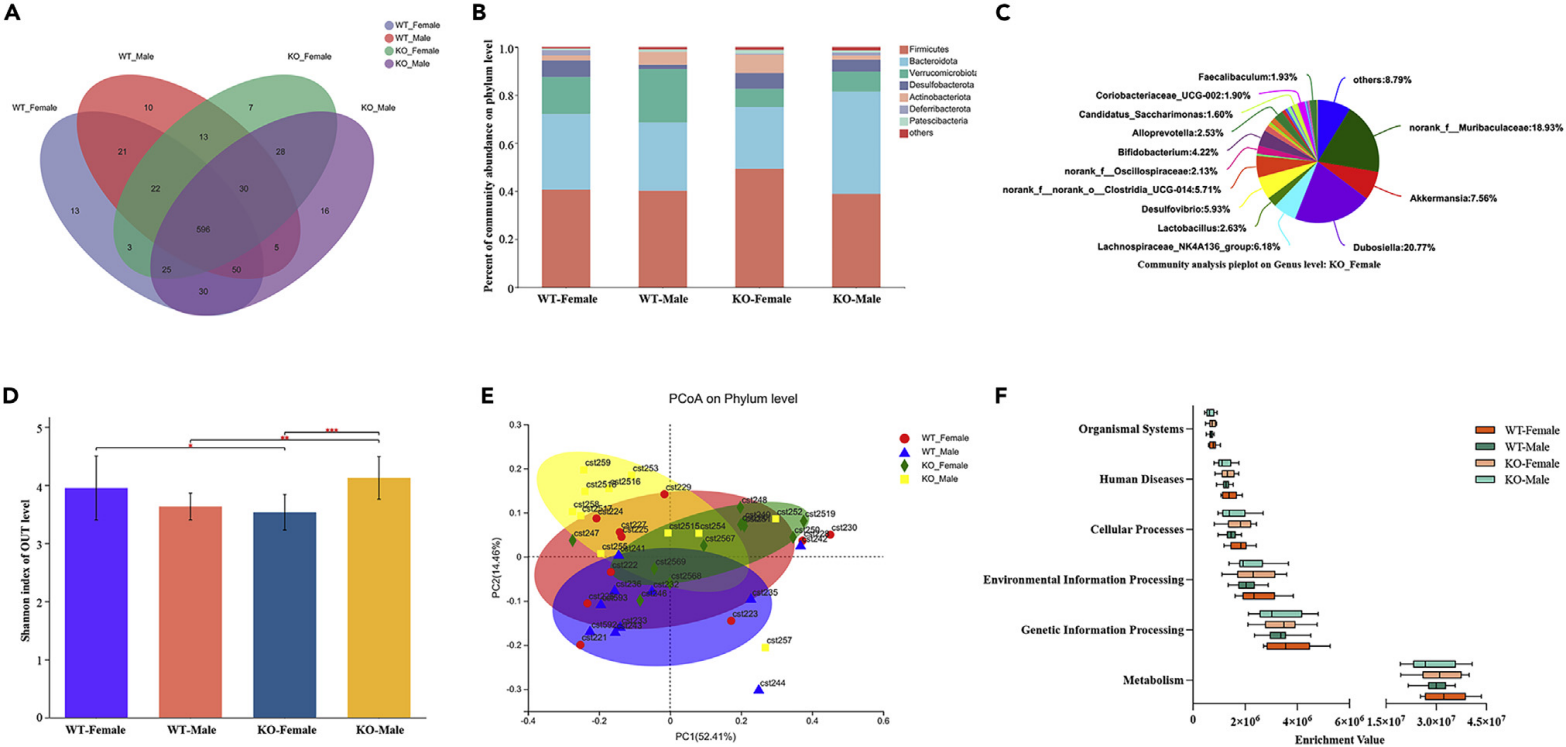
Mice with persistent HBV replication have altered gut microbiota community diversity and differentially abundant bacterial species (A) The Venn diagram shows the number of common and unique species (e.g., operational taxonomic units [OTUs]) in different groups. (B) Bacterial composition at the phylum level in the four groups. (C) Composition of gut microbiota in *ApoH*^*−/−*^ female mice at the genus level. (D) Alpha diversity of WT and *ApoH*^−/−^ mice with persistent HBV replication, as measured by the Shannon index. p < 0.05. (E) PCoA of beta diversity by comparing Bray Curtis distances to reveal the separation of microbial communities based on WT and *ApoH*^*−/−*^genotypes (p < 0.05). (F) Predictive functional profiling of microbial communities using PICRUSt and KEGG pathway enrichment.

We also explored the gut community diversity and bacterial species abundance in mice with persistently replicating HBV. The Venn diagram shows the number of common and unique species in different groups (Figure 7A). Figure 7B illustrates the bacterial composition at the phylum level in the four groups. We found that the abundance of *Verrucomicrobiota* sharply decreased in *ApoH*^*−/−*^ mice, and sex differences synergistically affected gut microbiota composition. The abundance of *Firmicutes*, *Actinobacteria*, and *Patescibacteria* had increased in female *ApoH*^*−/−*^ mice compared with that in female WT mice. Concurrently, the abundance of *Deferribacterota* and *Bacteroidota* had significantly decreased in female *ApoH*^*−/−*^ mice. However, among male mice, the abundance of *Deferribacterota* and *Bacteroidota* had observably increased in *Apoh*^*−/−*^ mice compared with that in WT mice, while the abundances of *Actinobacteria* and *Patescibacteria* had sharply decreased in *Apoh*^*−/−*^ mice.

We further analyzed the bacterial composition at the genus level among the four groups, and Figure 7C shows the detailed gut microbiota composition in *ApoH*^*−/−*^ female mice. A significant difference was observed in the alpha diversity between WT and *ApoH*^−/−^ mice and also between female and male mice in the *ApoH*^−/−^ group (p < 0.05) (Figure 7D). Next, the beta diversity assessment performed by principal coordinate analysis (PCoA) at the phylum level revealed a significant difference between the WT and *ApoH*^*−/−*^ groups (p < 0.05) (Figure 7E).

Finally, we used PICRUSt software to perform predictive functional profiling of microbial communities via KEGG enrichment analysis (Figure 7F). The gut microbiota mainly performed metabolic functions in *ApoH*^−/−^ and WT mice of different groups, but no significant difference was observed between groups (p > 0.05).

## Discussion

In the present study, we analyzed the serum APOH levels in patients with HBV-related chronic liver diseases and utilized the HepG2.2.15 cell line and an *ApoH*^*−/−*^plus HBV replication mouse model to further investigate the effect of APOH on hepatocyte steatosis and gut microbiota dysbiosis. The graphical abstract summarizes the key findings.

It has been known that APOH is an acute-phase protein in viral infection.^28^ In our previous study, we found that APOH is highly expressed in HepG2.2.15 cells, with HBV and the large surface antigen directly upregulating APOH expression.^24^ Therefore, we were intrigued to explore the role of APOH during the course of HBV infection. Firstly, we analyzed APOH levels in the sera of patients with chronic HBV infection and correlated with other biomarkers in HBV-related liver diseases. We found that the serum APOH levels were significantly decreased in patients with cirrhosis, and the reduction was more obvious in females than in males during the chronic infection phase. Further analysis indicated that the serum APOH levels were negatively correlated with the HBsAg levels and positively associated with the TG and albumin levels in patients with HBV-related chronic liver diseases. Taken together, we propose that APOH might play different roles in acute versus chronic HBV infection, and the potential sex-synergistic effect might be another hinge point between APOH and viral infection.

Next, on one hand, we confirmed that HBV significantly upregulated *APOH* expression in a non-temporal manner. We considered that APOH performs additional functions during chronic HBV infection, besides acting as an acute-phase protein in viral infection. On the other hand, we found that, during the course of HBV infection, APOH is switched from performing an early regulatory role in the immune function to a later regulatory role in metabolism. Further analysis indicated that some significant DEGs in APOH-silencing group, including *CYP21A2*, *CYP3A4, CYP11A1*, *CYP3A7*, and *AKR1810*, are associated with steroid biosynthesis and metabolism.^26^ Therefore, we speculate that the regulatory effect of APOH is associated with metabolic functions of liver in a gender-dependent manner with or without HBV infection.

In addition, patients suffering from chronic HBV infection with concomitant steatosis are at an increased risk of hepatic fibrosis, cirrhosis, and hepatocarcinoma.^8^^,^^9^ This motivates researchers to understand the molecular complexities associated with the diseases. Based on the current literature on the association of APOH and lipid metabolism,^15^^,^^16^^,^^17^^,^^18^^,^^19^^,^^20^ we believe that APOH protein polymorphism underlie variations in plasma lipid levels, as shown in Table S1. Moreover, we are interested in verifying the effect of APOH on lipid metabolism during chronic HBV infection in a future study.

*In vivo*, we also found that *ApoH*^−/−^ mice exhibited spontaneous steatohepatitis. Considering that gut-liver axis regulation and gut microbiota dysbiosis have been highlighted as crucial pathogenetic factors of NAFLD,^29^^,^^30^^,^^31^ here we further investigated the gut community diversity and the difference in bacterial species abundance in *ApoH*^−/−^ mice. The total bacterial composition and abundance at the phylum and genus levels had significantly decreased in *ApoH*^−/−^ mice, consistent with previous reports.^32^ Thus, it is likely that *ApoH* deletion in mice might induces dysbiosis in metabolism regulation and gut microbiota. We should have a better understanding of its role by constructing the non-alcoholic fatty liver mouse model in the background of *ApoH*^−/−^ mice.

Next, we constructed a persistent HBV replication mouse model in the background of *ApoH*^−/−^mice. During the periodic detection of serum HBsAg titers, we found that the serum HBsAg levels were higher in *ApoH*^−/−^ mice than in WT mice, which supports our previous finding from *in vitro* cell experiments, wherein high expression of APOH inhibited the HBsAg secretion.^25^ However, we found that the serum HBsAg, ALT, TG, and TC levels and the degree of fatty liver appeared to show sex-synergistic differences. It has been well documented that sex is a key factor in shaping the immune responses, contributing to differences in the pathogenesis of infectious diseases, between male and female patients.^33^^,^^34^ Moreover, metabolic homeostasis is differentially regulated between men and women, and there are sex-specific effects on lipid and cholesterol metabolism. Briefly, sexual differences exist in *ApoH* deletion-regulated lipid metabolic dysbiosis, HBsAg secretion, and the development of liver injury in chronic HBV infection.

Another factor in the development of fatty liver disease is the gut microbiota. Then, we detected the community diversity and the difference in bacterial species abundance in WT and *ApoH*^−/−^ mice on the background of persistent HBV replication. We found that *ApoH* deletion altered the community and diversity of the gut microbiota. The abundance of Verrucomicrobiota*,* an intestinal commensal microbiota in healthy individuals, had sharply decreased in *ApoH*^*−/−*^ mice. Second, sex differences affected the community and diversity of the gut microbiota. In the abundance of *Actinobacteria* and *Patescibacteria* versus *Deferribacterota* and *Bacteroidota*, an opposite trend was observed in *ApoH*^*−/−*^ female and male mice compared with that in WT mice. Third, the microbial communities were mainly associated with metabolic functions in different groups. Sex and gut microbiota have been reported to regulate host metabolism and viral infection. Figure S4 indicated the significant metabolic alterations in patients with chronic HBV infection. However, lipid metabolic dysbiosis and HBV-related liver injury also influence gut microbiota hemostasis.^10^^,^^35^^,^^36^ In addition, physiological processes are differentially regulated between men and women. Sex-specific *ApoH* deletion in mice promoted HBsAg secretion, disturbed lipid metabolism, and aggravated hepatocyte steatosis. Our on-going study should enhance our understanding of the regulatory mechanism.

Researchers have been trying to understand the molecular mechanisms underlying the development of CHB infection and NAFLD, and this study attempts to elucidate the pleiotropic effect of APOH in HBV-related chronic liver diseases. It provides a new perspective on sex-specific lipid metabolic dysbiosis by combining gut microbiota and HBV infection. This study provides a valuable framework to decipher the roles of APOH in chronic liver diseases. Obviously, in-depth studies on the mechanism of chronic liver diseases are crucial for clarifying the role of APOH in liver diseases, where animal models that can adequately reflect the clinical settings should be constructed.

### Limitations of the study

In our study, we had the unique and original opportunity to investigate the potential regulatory role of APOH during chronic HBV infection; this study, however, had some limitations. Firstly, the cross-sectional design and low sample size may limit the statistical power of APOH levels and correlation analysis. Then, we analyzed some open-source data deriving from the GEO databases to validate our findings (Figures S1 and S2). Next, in this study, we found *ApoH* deletion in mice might induce dysbiosis in metabolism regulation and gut microbiota. We should have a better understanding of its role by constructing the non-alcoholic fatty liver mouse model in the background of *ApoH*^−/−^ mice to pursue the further study on the association of *ApoH*-induced abnormality in metabolism and development of fatty liver and gut microbiota dysbiosis. In conclusion, we report unique effects of APOH in HBV-related chronic liver diseases. It provides a new perspective on sex-specific lipid metabolic dysbiosis by combining gut microbiota and HBV infection.

## STAR★Methods

### Key resources table

**Table:** 

REAGENT or RESOURCE	SOURCE	IDENTIFIER
**Bacterial and virus strains**		

rAAV8-1.3HBV, subtype ayw	Beijing Five-Plus Institute of Molecular Medicine Co. LTD	AMV-002

**Biological samples**		

Mouse liver tissue	This paper	N/A
Mouse feces	This paper	N/A

**Chemicals, peptides, and recombinant proteins**		

ON-TARGETplus Human APOH siRNA	Dharmacon^TM^, GE Healthcare	L-007811-00-0005
Lipofectamine^TM^ 3000 transfection reagent	Invitrogen	L3000008
Lipofectamine^TM^ RNAiMAX transfection reagent	Invitrogen	13778075

**Critical commercial assays**		

Human APOH ELISA Kit	Uscn Life Sciences Inc	SEA310Hu
RNeasy Mini Kit (50)	QIAGEN	74104
Oil Red O Stain Kit (Lipid Stain)	Abcam	ab150678
ALT	Mindray	105-000442-00
AST	Mindray	105-000443-00
TG	Mindray	105-000449-00
TC	Mindray	105-000448-00

**Deposited data**		

Raw and analyzed data	This paper	GEO: GSE65359, GSE84044, GSE162694
Raw and analyzed data (serum metabolic alterations in patients with chronic HBV infection)	This paper	https://www.frontiersin.org/articles/10.3389/fmicb.2017.02222/full#supplementary-material

**Experimental models: Cell lines**		

HepG2.2.15	Academy of Military Medical Sciences (Beijing, China)	Gifted from Dr. Shengqi Wang
HepG2-NTCP	This paper	N/A

**Experimental models: Organisms/strains**		

ApoH gene knockout mouse model	This paper	N/A
HBV replicative mouse model	This paper	N/A

**Oligonucleotides**		

siRNA targeting sequence: APOH	This paper	N/A
Primers for DEGs, see Table 2	This paper	N/A

**Recombinant DNA**		

Plasmid: VR1012-APOH	This paper	N/A

**Software and algorithms**		

PICRUSt software	This paper	https://github.com/picrust/picrust

### Resource availability

#### Lead contact

Further information and requests for resources and reagents should be directed to and will be fulfilled by the lead contact, Dr. Yaming Liu (yaming08565@gmail.com).

#### Materials availability

This study did not generate unique reagents.

### Experimental model and subject details

#### Animal care and generation of ApoH^−/−^ mice

All experiments on mice were approved by the Institutional Animal Care and Use Committee of Xiamen University (XMULAC20210119) and were conducted in accordance with institutionally approved protocols and guidelines for animal care and use. The detail protocol for the generation of the *ApoH*^-/-^ mouse model was described in our previous publication.^37^

#### Mouse model of persistent HBV replication

The HBV replication mouse model was constructed using six- to seven-week-old female and male mice that were either *ApoH*^*-/-*^ or WT controls. First, HBV plasmids were purchased from the Beijing Five-Plus Institute of Molecular Medicine Co. LTD. The HBV plasmid was derived from a recombinant adeno-associated virus type 8 with 1.2 times the HBV genome (rAAV8-1.3HBV, subtype ayw). Theres plasmids were then injected into the tail vein of the mice by hydrodynamic injection (5 × 10^10^ μg/200 μL per mouse). After injuection, orbital venous blood was collected weekly for 9 weeks, and the titer of HBsAg was measured using enzyme-linked immunosorbent assay (ELISA).

#### Patient samples

Serum samples from 53 patients with chronic HBV infection and 18 healthy controls were used in this study. The samples were collected from the First Hospital of Jilin University in Changchun, China. Patients with chronic infection, chronic hepatitis, and cirrhosis were diagnosed^38^ and treated with antiviral drugs in an outpatient setting. All procedures in this study were performed in accordance with the ethical standards of the medical ethics committee of the Zhongshan Hospital Xiamen University, and the First Hospital of Jilin University.

#### HepG2.2.15 cell line and culture

HepG2.2.15, a stable HBV-producing human hepatoblastoma cell line, was obtained from the Academy of Military Medical Sciences (Beijing, China). The cells were cultured in Dulbecco’s modified Eagle’s medium (DMEM; Hyclone, Waltham, MA, USA) supplemented with 10% fetal bovine serum (FBS), 1% penicillin/streptomycin (Gibco, Grand Island, NY, USA), and 380 μg/ml Geneticin® G418 sulfate (Gibco) in a 5% CO_2_ humidified atmosphere.

### Method details

#### ELISA for APOH in human serum

Serum samples were drawn from individuals using sterile syringes and stored individually in blood collection tubes to avoid cross-contamination. The serum samples were kept at −80°C until used for analysis. The serum APOH levels (40,000-fold dilution) were quantified using a commercial ELISA kit (Uscn Life Science Inc, Wuhan, China) according to the manufacturer’s instructions.

#### HBV infection

The detail procedure for HBV infection has been previously described.^39^ Briefly, HepG2-NTCP-tet cells were cultured with 4 μg/mL doxycycline (DOX) for 4 days to induce NTCP expression. The cells were then seeded in a 24-well plate and maintained in DMEM for 24 h. The supernatant of HepAD38 cells was concentrated using 6×PEG8000 buffer (48% PEG8000 and 200 mM NaCl). HepG2/NTCP cells were then inoculated in the presence of 4% PEG8000 and 2% dimethyl sulfoxide for 24 h. The cells were washed six times with phosphate-buffered saline and maintained in DMEM. The medium was changed every 2 days.

#### HepG2.2.15 cell transfection

For transfection experiments, the cells were seeded in 6-well plates and cultured for 24 h to reach 50–60% confluence on the day of transfection. Transient transfections were performed using Lipofectamine^TM^ 3000 transfection reagent (Invitrogen, Waltham, MA, USA) in accordance with the manufacturer’s instructions.

For small interfering RNA (siRNA)-mediated interference, HepG2.2.15 cells in 6-well cell culture plates were transfected with 30 pM siRNA using Lipofectamine RNAiMAX (Thermo Fisher Scientific, Waltham, MA). After incubation for 24 h, the cells were washed and used for the experiments. The siRNAs specific for mouse *ApoH* or human *APOH* mRNA (SMART pool) and non-targeting siRNAs were purchased from Dharmacon (GE Healthcare).

#### Real-time qPCR

An RNeasy kit (QIAGEN, Germantown, MD, USA) was used to extract the total RNA from the cells according to the manufacturer’s instructions. Detailed procedures were described in a previous report.^25^ *GAPDH* was amplified as an internal control in the same reaction per sample. The relevant qPCR primers were listed in below Table. Each experiment was performed in triplicate. The mRNA levels were calculated using the 2^−ΔΔCt^ method and normalized to *GAPDH* mRNA levels.

**Table:** Primers used in RT-qPCR analysis

Gene	Forward Primers (5′- 3')	Reverse Primers (5′- 3')
Mouse GAPDH	AACGACCCCTTCATTGAC	TCCACGACATACTCAGCAC
Mouse ApoH	TGGCATTGAACTCACACT	GAATGTTCCTGGCAGTTG
Human GAPDH	AATCCCATCACCATCTTCCA	TGGACTCCACGACGTACTCA
Human APOH	GCCCATCAACACTCTGAAATG	CGCCATTCAGATAAAACCCAG
HBV *S* region	ATGGAGAACATCACATCAGG	TTAAATGTATACCCAAAGACAAAAG
Human CYP2C9	CAGAGACGACAAGCACAACCCT	ATGTGGCTCCTGTCTTGCATGC
Human CYP3A4	CCGAGTGGATTTCCTTCAGCTG	TGCTCGTGGTTTCATAGCCAGC
Human CYP3A7	GTCCCTATCATTGCCCAGTATG	AGTCGATGCTCACTCCAAATG
Human CYP7A1	AATTTCACTTTGCTACTTCTGCG	TGAGGGAATTCAAGGCATGG
Human CYP11A1	TGGCATCCTCTACAGACTCCTG	CTTCAGGTTGCGTGCCATCTCA
Human CYP21A2	GTGGTGCTGAACTCCAAGAGGA	GAGTAGTCTCCCAAGGACAGGT
Human CYP26A1	AGCTGTTGATCGAGCACTCGTG	GGTTTCGTGTCCTCCAAAGAGG
Human NT5E	AGTCCACTGGAGAGTTCCTGCA	TGAGAGGGTCATAACTGGGCAC
Human AKR1B10	GAGGACCTGTTCATCGTCAGCA	CGTCCAGATAGCTCAGCTTCAG

#### RNA-seq

The detailed protocol had been described previously.^25^ Briefly, HepG2.2.15 cells were seeded in 6-well plates and cultured for 24 h. In HepG2.2.15 cells, an siRNA was used to silence *APOH* expression, and an *APOH* plasmid was transfected for overexpression. After 48 h post-transfection, we collected the cells and extracted the total RNA using an RNeasy kit (QIAGEN) according to the manufacturer’s instructions. These were repeated three or more times, and then the selected samples were sent to The Beijing Genomics Institute (BGI) for RNA-seq (N = 2). The phyper function of the R software was used to perform enrichment analysis, and a FDR-adjusted p-value (Q-value) ≤ 0.05 represented significant enrichment. In the Kyoto Encyclopedia of Genes and Genomes (KEGG) pathway analysis, the X-axis represents the enrichment ratio of candidate genes over total genes for a given term, and the Y-axis shows the KEGG pathway.

#### Assaying ALT and AST levels in mouse serum

Plasma samples were collected by centrifuging mouse peripheral blood at 3000 rpm for 15 min. The supernatants were kept at −80°C until used for analysis. The serum ALT and AST levels were quantified using a Mindray fully automatic biochemical analyzer (BS-240vet).

#### Histopathological analysis

Mouse liver sections were stained with hematoxylin and eosin (H&E) or Oil Red O (Abcam, Cambridge, MA, USA) and observed under a fully automatic biological microscope (Motic® BA600-4, Fujian, China).

#### Quantitative analysis of 16S rDNA genes

Prior to euthanization, stool samples were collected from all mice, and the samples were sent to Majorbio Corporation to detect microbial diversity. The V3-V4 region of the 16S rRNA gene was used as the bacterial-specific fragment and amplified with the primers 338F (5′-ACTCCTACGGGAGGCAGCAG-3′) and 806R (5′-GGACTACHVGGGTWTCTAAT-3′). Amplicons were sequenced using the Illumina HiSeq platform (Illumina, San Diego, CA, USA) and analyzed with an automatic analysis software from Majorbio. Additional analysis and data visualization were performed in R 3.6.1. Microbial community diversity was measured using the Shannon–Weiner biodiversity index (Shannon index). The potential function of microbial communities from sequencing data was analyzed using PICRUSt software (https://github.com/picrust/picrust), followed by MetaCys and/or KEGG pathway analysis.

### Quantification and statistical analysis

Due to the skewed distribution of data from human samples, the Kruskal-Wallis test, a non-parametric method, was used to identify differences between diagnostic groups, followed by the Mann–Whitney *U* test (two-tailed). Correlations between two variables were tested using Spearman rank correlation. Statistical significance was set at *p*<0.05.

Experimental results are expressed as mean ± SEM from three or more independent experiments. Two-tailed Student’s *t*-test was used, as appropriate, to test for significant differences between groups. Differences were considered significant at *p*<0.05.

## Data Availability

Data statement: The datasets generated and analyzed during the current study in the NCBI SRA repository (http://submit.ncbi.nlm.nih.gov/subs/sra/SUB12483444). The clinical trial registry and trial identifying number is xmzsyyky2020145 (http://114.255.48.20). All data related to this study are available upon request. Code statement: This paper does not report original code. The source and identifier of analysis code used in the paper can be found in the ‘key resources table’ and/or ‘quantification and statistical analysis’ section. Any additional information required to reanalyze the data reported in this paper is available from the lead contact upon request.
