# Supplementary material for: Apolipoprotein H induces sex-specific steatohepatitis and gut dysbiosis during chronic hepatitis B infection
Source: iScience. 2023 Feb 1;26(3):106100. doi: 10.1016/j.isci.2023.106100 (PMC9958358; doi:10.1016/j.isci.2023.106100)
Supplement: Document S1. Figures S1–S4 and Tables S1 and S2 [file mmc1.pdf]

iScience, Volume *26*

Supplemental information

Apolipoprotein H induces sex-specific steatohepatitis and gut dysbiosis during chronic hepatitis B infection

Yaming Liu, Yangtao Wu, Xiaoming Jiang, Bo Chen, Jing Lu, Zexin Cai, Baorong Fu, Wei Zheng, Ruihong Wu, Gang Chen, Shulan Tian, and Jianlin Ren

Figure S1. Analysis of APOH levels between patients with chronic HBV infection

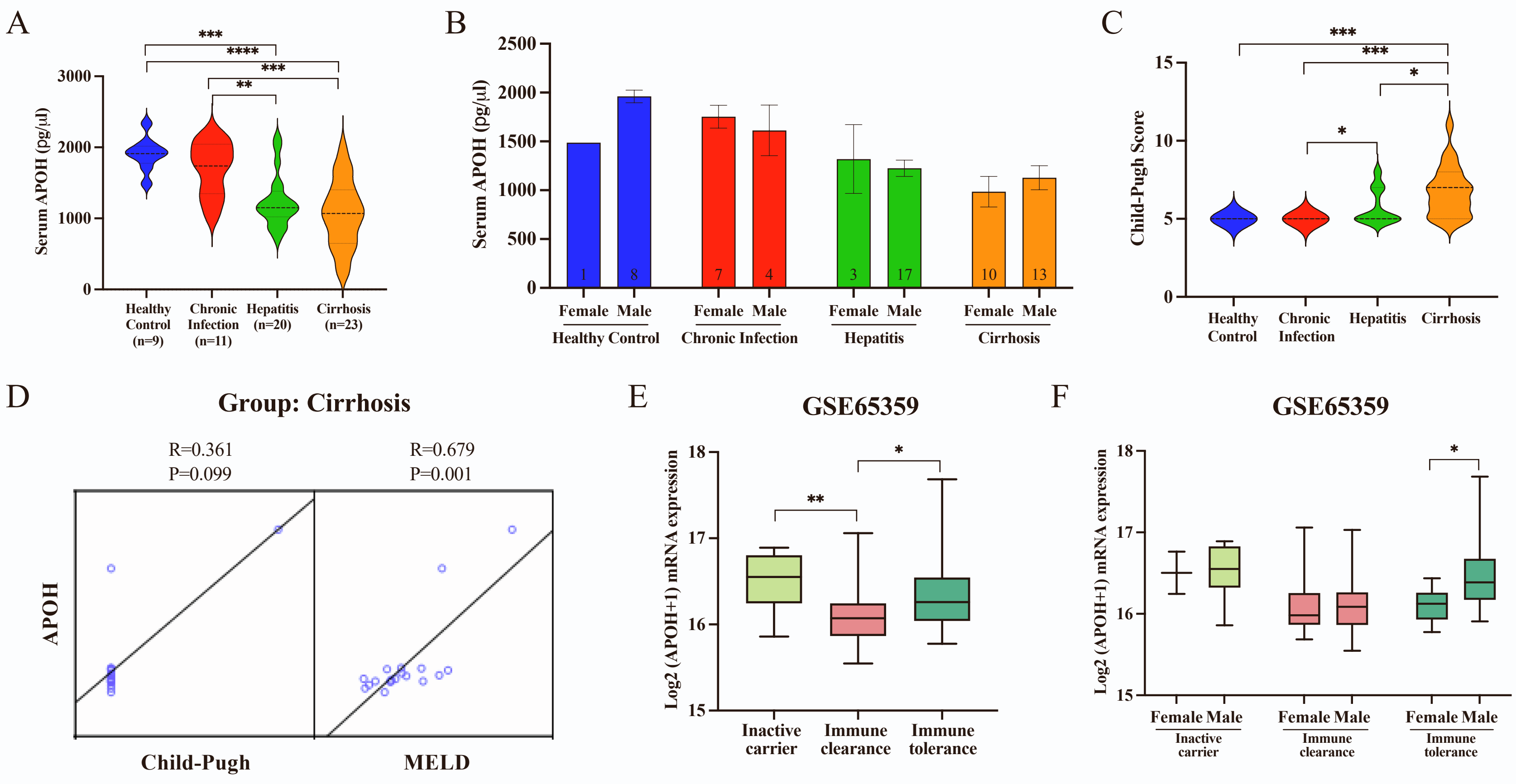

Figure S2. ***APOH*** **expression varies with the liver fibrosis stage in patients with chronic HBV infection or non-alcoholic fatty liver disease (NAFLD)**

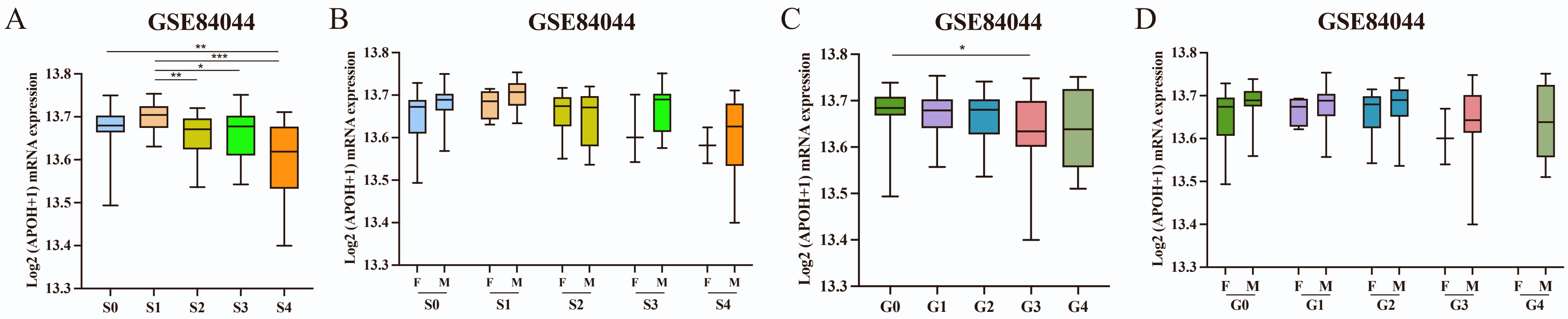

(Continuous)

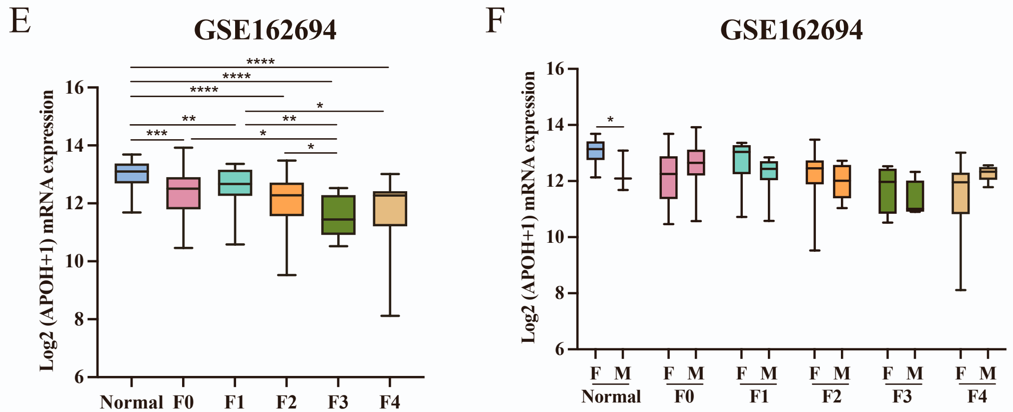

Figure S3. Serum HBsAg levels in *ApoH*$^{-/-}$ mice with persistent HBV replication

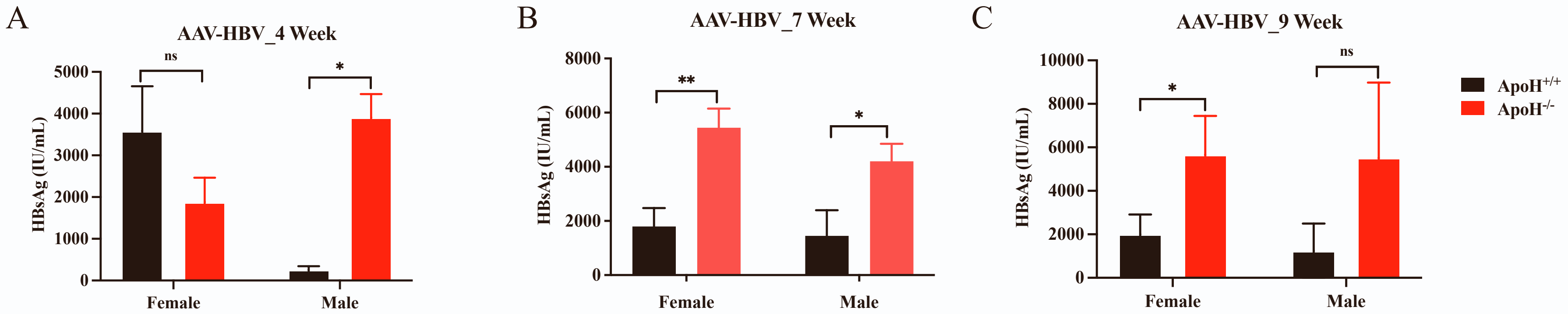

Figure S4. Significant metabolic alterations in patients with chronic HBV infection

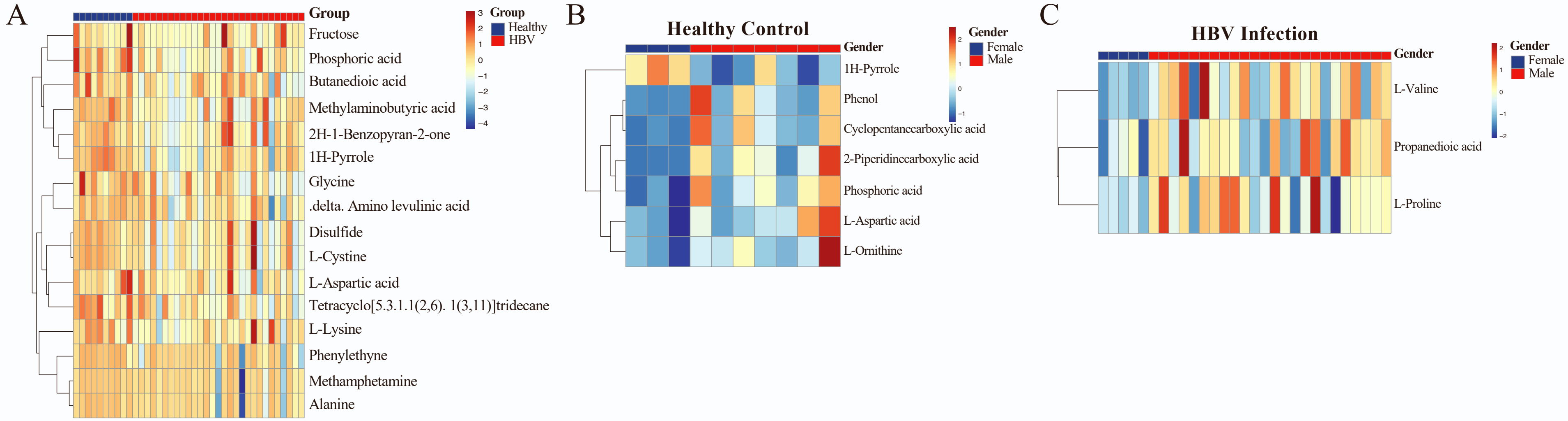

Table S1. ApoH protein polymorphism underlying variations in plasma lipid levels

APOH genotype	exon	Position	SNP	Mutation	Codon	Amino acid	Function
**H*1**	3	3333[a]	rs8178833	G→A	88	Ser→Asn	Increased HDL-C and apoA-I levels
**H*2**	7	14749	rs4791077	T→G	306	Cys→Gly	Reduced TG and apoE levels
**H*3**	5	8643[a]	rs1803122	T→C	122	Ile→Thr	Reduced TC, LDL-C, and apoB levles
**H*3**	5	8682[a]	rs8178847	G→A	135	Arg→His	Reduced TC, LDL-C, and apoB levles
**H*3/3[W]**	8	17212	rs1801690	G→C	316	Trp→Ser	Reduced TC, LDL-C, and apoB levles
**H*3/3[B]**	5	8700[a]	reference	C→A	141	Ala→Asp	Reduced TC, LDL-C, and apoB levles

SNP: single-nucleotide polymorphism. [a]SNPs that determine known apoH protein charge isoforms.

Table S2. Classification of human CYP enzymes based on P450-dependent diseases

P450-dependent Diseases	CYP enzymes
Steroid biosynthesis and metabolism	1B1, 7A1, 7B1, 8B1, 11A1, 11B1, 11B2, 17A1, 19A1, 21A2, 27A1, 39A1, 46A1, 51A1
Cholesterol biosynthesis and metabolism	51, 7A1, 7B1, 27A1, 46A1
Activation and inactivation of Vitamin D3	2R1, 24A1, 26A1, 26B1, 26C1, 27B1
Eicosanoid synthesis	4A11, 4A22, 4F2, 4F3, 4F8, 5A1, 8A1
Xenobiotics metabolism	1A1, 1A2, 2A6, 2A13, 2B6, 2C8, 2C9, 2C18, 2C19, 2D6, 2E1, 2F1, 3A4, 3A5, 3A7
Fatty acids biosynthesis	2J2, 4A11, 4B1, 4F12
